# Association between changes in body fat and disease progression after breast cancer surgery is moderated by menopausal status

**DOI:** 10.1186/s12885-017-3869-1

**Published:** 2017-12-18

**Authors:** Li-Ni Liu, Yung-Chang Lin, Christine Miaskowski, Shin-Cheh Chen, Mei-Ling Chen

**Affiliations:** 1 Department of Nursing, Fu Jen Catholic University, New Taipei City, Taiwan; 2 Department of Internal Medicine, Chang Gung Memorial Hospital and Chang Gung University, Taoyuan, Taiwan; 3 Department of Physiological Nursing, School of Nursing, University of California, San Francisco, USA; 4 Department of Surgery, Chang Gung Memorial Hospital and Chang Gung University, Taoyuan, Taiwan; 5 School of Nursing, College of Medicine, Chang Gung University, No.259, Wenhua 1st Rd., Guishan Dist., Taoyuan City, 33302 Taiwan, Republic of China; 6 Department of Nursing, Chang Gung University of Science and Technology, Taoyuan, Taiwan

**Keywords:** Breast cancer, Menopausal status, Body fat, Disease progression

## Abstract

**Background:**

Obesity is linked to poor disease outcomes in breast cancer patients. However, this link was mostly based on body weight or BMI rather than body-fat. The purpose of this study was to evaluate the relationship between body-fat gain and disease progression in Taiwanese women after breast cancer surgery and how this relationship is influenced by menopausal status.

**Methods:**

Body fat percentage was measured 1 day before and 6 months after surgery in 131 women with stages 0–III breast cancer. Disease outcomes (metastasis and death) were assessed by chart review and telephone contact 7 to 8 years after diagnosis. These data were analyzed by multivariate Cox proportional hazard model analysis.

**Results:**

The percentage of women with over 5% gain in body-fat was 56% for premenopausal and 42% for postmenopausal. Rates of distant metastasis and all-cause mortality were 17.6 and 9.9%, respectively over the follow-up period. Distant metastases were predicted in postmenopausal but not premenopausal women with breast cancer by increased body fat percentage (HR = 1.3, *p* = 0.035), after controlling other potential covariates, including disease severity, estrogen receptor expression, progesterone receptors expression, age, and exercise habit before diagnosis. Survival was not significantly associated with body-fat percentage gains.

**Conclusions:**

Our results suggest that increased body fat percentage 6 months after breast surgery is an important predictor of distant metastasis in postmenopausal Taiwanese women with breast cancer. Clinicians may need to measure patients’ body fat periodically. Our findings should be validated in studies with a longer follow-up time.

## Background

Breast cancer is the most common female cancer in Taiwan, with more than 10,000 women diagnosed every year [1]. Breast cancer has a relatively high 5-year survival rate of 86.5% [2], compared to the survival rate of 53.3% for all cancers combined. Despite this good prognosis, most breast cancer survivors who have completed treatment are still concerned about recurrence, metastasis, or mortality [3]. Predictive factors for poor outcomes include disease stage, axillary lymph node involvement, negative expression of estrogen receptors (ER) and progesterone receptors (PR), younger age [4], and exercise [5]. In addition, weight gain was reported to be a risk factor for poor breast cancer outcomes [6–9].

Weight gain during or after breast cancer treatment due to changes in metabolism, physical activity, and dietary intake [10] is well documented. After treatment, 34% to 96% of women with early-stage breast cancer gain 0.9–7 kg (kg) [11–15] and they do not automatically lose this extra weight when treatment ends [16, 17].

Besides gaining weight, these women experience significant increases in the percent of body fat [13, 18, 19]. However, body weight or body mass index (BMI), which can be easily measured, are used in most studies as a surrogate measure for obesity [13, 20]. Since body weight fails to discriminate between body fat and lean mass [21, 22], changes in body fat cannot be precisely ca ptured by measuring body weight [10, 13, 14]. Body fat, as the main body component inducing higher amounts of circulating estrogens, interleukin 6, tumor necrosis factor alpha, retinol-binding protein-4, C-reactive protein and leptin, may be a more appropriate indicator to monitor than BMI or body weight for its impact on disease outcomes [10, 23–25]. However, no study has elucidated the effect of body fat change after diagnosis on breast cancer outcomes.

In determining the relationship between obesity and occurrence of breast cancer, several lines of evidence suggest an influential role for menopausal status. For example, obesity increases breast cancer risk after menopause but decreases this risk before menopause [13, 26, 27]. In addition, premenopausal women with breast cancer had more weight gain after treatment than did postmenopausal women [16, 28]. It is hypothesized that menopausal status may modify the effect of body fat gain on disease progression in women with breast cancer.

Therefore, this study had two purposes: (1) to evaluate the relationship between body-fat percentage gain and disease progression in Taiwanese women after breast cancer surgery; and (2) to explore the role of menopausal status in moderating the relationship between body-fat percentage change and disease progression.

## Methods

### Design, setting, and sample

This is a prospective observational study. The sample for this study was drawn from a longitudinal study that examined symptoms after surgery in newly diagnosed breast cancer patients who were treated at Chang Gung Memorial Hospital (Linkuo and Taipei), a large teaching hospital treating cancer patients from all parts of Taiwan. Patients’ inclusion criteria have been published [29]. In brief, women with stage 0-III breast cancer who underwent unilateral breast cancer surgery were included. Women were excluded if they had breast cancer surgery on both sides, had distant metastasis at diagnosis, and/or had a defibrillator implanted. Of the 239 women invited to participate between July 2005 and September 2006, 200 provided written informed consent and were enrolled. In the original study, demographic and exercise habit data were acquired at baseline (the day prior breast surgery). Treatment information was collected at 6 months after breast surgery by reviewing medical charts. Anthropometric measures were assessed at baseline and 6 months after surgery. In 2013 (i.e., 7 to 8 years after diagnosis), we further determined these women’s disease outcomes (distant metastasis and all-cause mortality) by reviewing medical charts. If the patient did not return to the hospital for more than 3 months and her survival status was not documented in medical chart, a telephone call was made to the patient or her primary caregiver to confirm the survival and metastasis status. Among these 200 women, 131 (65.5%) had complete data for both anthropometric measures and disease progression outcomes. The data from these 131 women were analyzed in this study. For those who had missing data (*n* = 69), 63 had missing data on body fat information at 6 months, 10 on distant metastasis information, and 6 on all-cause mortality information. Patients who had missing data on anthropometric measures was mainly because they did not come to the hospital for anthropometric measures if no treatment or follow-up was scheduled at 6 months after surgery. Compared to those with valid data, those with missing data were found to be older and had higher percentages of not receiving any chemotherapy or radiotherapy. Other variables (including initial BW, initial BMI, initial fat percentage, exercise habit, the distribution of disease stage, ER/PR status, hormonal therapy, distant metastasis, and all-cause mortality) were comparable between those with valid data and those with missing data (*p* > 0.05). This study was approved by the Institutional Review Board of Chang Gung Memorial Hospital.

### Measurements

#### Anthropometry

Participants’ height and weight were measured using a digital electronic professional scale with height rod (Super-View Medical: HW686, Taiwan). BMI is calculated as weight (in kg) divided by height (in meters (m)) squared. Body fat percentage is calculated as body fat (in kg) divided by body weight (in kg). Body fat was measured with a portable hand-to-foot tetrapolar RJL Quantum-X Bioelectrical Impedance Analyzer (BIA) machine (RJL Systems, Clifton Township, MI, USA). BIA has been reported as easy to use, free from radiation exposure, and having good agreement in detecting body composition compared to dual X-ray absorptiometry (DXA) [30]. Given that different body tissues have different levels of electrical conductivity, it will not be detected in all tissues when a small alternating current (500 microamps at a single frequency of 50 kHz) is applied through the body. The extent to which the applied current encounters resistance or impedance is related to the type and amount of tissue through which the current passes. For example, fat tissue has higher impedance than muscle tissue [31, 32]. Based on this property, body composition can be estimated with the software Cyprus 2.7. To obtain the BIA measurement, patients removed their shoes and socks from the foot of their non-operated side and lay in a supine position on an examination table with their legs apart and arms abducted from the body. Four self-adhesive spot electrodes were placed on the dorsal surface of the hand and foot of the non-operated side. To minimize measurement error, patients were told not to eat or drink too much and to refrain from alcohol intake and exercise for 8 h before this measurement [32]. Anthropometric measurements were made by a trained research assistant.

#### Disease progression

Data were collected from medical chart review and telephone interviews with patients from April to June 2013 about two disease-progression events: distant organ metastasis induced by breast cancer and all-cause mortality. Approximately 15% of the disease progression data were obtained through telephone interviews. For distant metastasis or death, time to the event was defined as months between the date of cancer diagnosis and date of the event.

#### Covariates

Demographic and disease/treatment information were collected using a researcher-developed form. Women were considered menopausal if they reported that their menses had stopped for more than 6 months [33]. Disease stage was re-categorized as a binary variable, disease severity: low severity (stage 0-II) and high severity (stage III). The exercise habit was a yes/no categorical variable assessed at baseline. Patients were asked whether they exercised on a regular basis during the past month. Covariates included for analysis were disease stage, ER status, PR status, age, and exercise habit. Treatment modality was not included because it was highly associated with disease stage.

### Statistical analysis

The distribution of study variables was described by means, standard deviations (SD), and percentages. Changes in anthropometric variables were defined by subtracting their values before surgery from those 6 months post-surgery. Differences in demographic, exercise habit, anthropometric, disease, treatment, and disease progression characteristics between premenopausal and postmenopausal groups were evaluated by t-test for continuous variables and chi-square test for categorical variables. The effect of body fat percentage change and its interaction with menopausal status on disease progression were determined by multivariate Cox proportional hazard model analysis. The main effect of body fat percentage change adjusted for 5 covariates (disease stage, age, ER, PR, and exercise habit) was first tested. Then, the overall interaction effect of body fat percentage changes and menopausal status was examined. When a significant interaction was found, subgroup analysis was then performed for pre- and post-menopausal groups separately. All analyses were performed using SPSS software, version 19.0 (Chicago, IL). Significance levels were set at 0.05 for two-tailed tests.

## Results

### Patients’ characteristics

Among the 131 women in our sample, 86 (65.6%) were premenopausal and 45 (34.4%) were postmenopausal at diagnosis. Their average age was 46.9 years. Most patients did not exercise regularly (72.5%) and had stage II or earlier disease (80.9%), with 69.5% ER positive and 55.0% PR positive. More than half of the women received breast modified radical mastectomy (MRM) surgery (56.5%). Most women received adjuvant chemotherapy (83.2%) while 55.0% received radiotherapy and 63.4% received hormone therapy. Compared to premenopausal women, postmenopausal women were older (56.1 vs. 42.1 years), more of them were PR negative (66.3 vs. 33.3%), and fewer received radiotherapy (40.0 vs. 62.8%) (Table 1).

**Table 1:** Demographic, exercise habit, and disease/treatment information by menopausal status in women with breast cancer

Characteristic	Total	Premenopausal	Postmenopausal	*t / χ* ^2^	*p*
	(*n* = 131)	(*n* = 86)	(*n* = 45)		
	mean ± SD	mean ± SD	mean ± SD		
	*n* (%)	*n* (%)	*n* (%)		
Age (years)	46.9 ± 9.5	42.1 ± 7.0	56.1 ± 6.3	11.2	< 0.001
Exercise habit before diagnosis				3.6	0.056
No	95 (72.5%)	67 (77.9%)	28 (62.2%)		
Yes	36 (27.5%)	19 (22.1%)	17 (37.8%)		
Type of surgery				12.6	< 0.001
BCT	57 (43.5%)	47 (54.7%)	10 (22.2%)		
MRM	74 (56.5%)	39 (45.3%)	35 (77.8%)		
AJCC staging				0.8	0.664
Stage 0/ I	50 (38.2%)	35 (40.7%)	15 (33.3%)		
Stage II	56 (42.7%)	36 (41.9%)	20 (44.4%)		
Stage III	25 (19.1%)	15 (17.4%)	10 (22.3%)		
Estrogen receptor				2.9	0.089
Positive	91 (69.5%)	64 (74.4%)	27 (60.0%)		
Negative	40 (30.5%)	22 (25.6%)	18 (40.0%)		
Progesterone receptor				13.0	< 0.001
Positive	72 (55.0%)	57 (66.3%)	15 (33.3%)		
Negative	59 (45.0%)	29 (33.7%)	30 (66.7%)		
Chemotherapy				0.5	0.478
Yes	109 (83.2%)	73 (84.9%)	36 (80.0%)		
No	22 (16.8%)	13 (15.1%)	9 (20.0%)		
Radiotherapy				6.2	0.013
Yes	72 (55.0%)	54 (62.8%)	18 (40.0%)		
No	59 (45.0%)	32 (37.2%)	27 (60.0%)		
Hormone therapy				3.0	0.085
Yes	83 (63.4%)	59 (68.6%)	24 (53.3%)		
No	48 (36.6%)	27 (31.4%)	21 (46.7%)		
Distant organ metastasis				1.0	0.310
Yes	23 (17.6%)	13 (15.1%)	10 (22.2%)		
No	108 (82.4%)	73 (84.9%)	35 (77.8%)		
All-cause death				0.9	0.345
Yes	13 (9.9%)	7 (8.1%)	6 (13.3%)		
No	118 (90.1%)	79 (91.9%)	39 (86.7%)		

*AJCC* American Joint Committee on Cancer, *SD* standard deviation, *BCT* Breast Conserving Therapy, *MRM* Modified Radical Mastectomy

#### Anthropometric measures by menopausal status

The mean BMI before surgery was 23.1 kg/m^2^ for premenopausal women and 24.5 kg/m^2^ for postmenopausal women. The mean body fat before surgery was 17.9 kg for premenopausal women and 20.0 kg for postmenopausal women. For BMI, both groups had similar percentages (around 31%) for >5% gain at 6 months after surgery. However, the percentages of >5% gain in body fat were higher than that of BMI for both premenopausal (56%) and postmenopausal (42%) groups. Premenopausal and postmenopausal groups did not differ significantly in the percentages of >5% gain for all the anthropometric variables (Table 2).

**Table 2:** Changes in anthropometric variables by menopausal status in women with breast cancer (*N* = 131)

	Premenopausal (*n* = 86)			Postmenopausal (*n* = 45)				
Anthropometry	Presurgery (M ± SD)	Post- surgery^a^ (M ± SD)	Gain >5% *n* (%)	Presurgery (M ± SD)	Post-surgery^a^ (M ± SD)	Gain >5% *n* (%)	*χ* ^*2*^	*p*
Weight (kg)	56.5 ± 8.2	58.3 ± 8.2	27 (31.4%)	58.1 ± 9.2	59.1 ± 8.3	14 (31.1%)	< 0.1	0.973
BMI (kg/m^2^)	23.1 ± 3.0	23.8 ± 3.1	27 (31.4%)	24.5 ± 3.3	24.9 ± 2.9	14 (31.1%)	< 0.1	0.973
Body fat (kg)	17.9 ± 5.8	19.1 ± 6.0	48 (55.8%)	20.0 ± 6.1	20.4 ± 5.5	19 (42.2%)	2.2	0.139
Body fat (%)	31.0 ± 6.4	32.1 ± 6.3	36 (41.9%)	33.7 ± 6.3	33.9 ± 5.6	14 (31.3%)	1.4	0.229

*BMI* body mass index, *M* mean, *SD* standard deviation

^a^Six months after surgery

#### Effect of body fat percentage change on disease progression

The median period between diagnosis and chart review was 7.6 years (range = 7–8.1). During this period, 23 (17.6%) patients developed distant organ metastasis and 13 (9.9%) died. The censored rates for distant organ metastasis and for all-cause mortality were 82.4 and 90.1%, respectively. Before examining the effect of body fat percentage change, we tested the impact of BMI change on study outcomes and found that after adjusting the covariates, no significant main effect of BMI change on both distant metastasis (*B* = 0.35, *p* = 0.058) and all-cause mortality (*B* = 0.11, *p* = 0.066). We then examined the effect of body fat percentage change on study outcomes and also found no significant effect on distant metastasis (*B* = 0.11, *p* = 0.063) or all-cause mortality (*B* = 0.13, *p* = 0.104). However, disease stage was found to be a significant predictive factor for metastasis and mortality (*p* < 0.001) (Table 3).

**Table 3:** Effects of body fat percentage change on distant metastasis and all-cause mortality in women with breast cance (*N* = 131)

	*B (± SE)*	Hazard ratio (95% CI)	*p*
Metastasis			
Advanced disease (Stage III)	1.90 (± 0.45)	6.71 (2.78–16.17)	< .001
ER positive	0.82 (± 0.56)	2.26 (0.75–6.84)	.148
PR positive	−0.91 (± 0.52)	0.40 (0.15–1.11)	.080
Age	−0.02 (± 0.03)	0.98 (0.93–1.03)	.372
Regular exercise	0.06 (± 0.53)	1.07 (0.38–3.00)	.905
% body fat change	0.11 (± 0.06)	1.11 (0.99–1.25)	.063
Death			
Advanced disease (Stage III)	2.97 (± 0.69)	19.49 (5.10–74.56)	< .001
ER positive	0.41 (± 0.74)	1.51 (0.36–6.38)	.576
PR positive	−0.98 (± 0.69)	0.37 (0.10–1.45)	.155
Age	0.02 (± 0.04)	1.02 (0.95–1.10)	.537
Regular exercise	−0.30 (± 0.72)	0.74 (0.18–3.05)	.676
% body fat change	0.13 (± 0.08)	1.14 (0.97–1.33)	.104

Body fat % change = body fat percentage at 6 months after surgery minus body fat percentage before surgery

*ER* estrogen receptor, *PR* progesterone receptor

#### Interaction effect of body fat percentage change and menopausal status on disease progression

Similar to the analysis of main effect, we first checked the interaction effect between BMI change and menopausal status and no significant interaction was found for both distant metastasis (*B* = 0.62, *p* = 0.071) and all-cause mortality (*B* = 0.53, *p* = 0.126). However, after adjusting for covariates, the interaction effects of body-fat percentage change and menopausal status on both distant metastasis (*B* = 0.29, *p* = 0.012) and all-cause mortality (*B* = 0.32, *p* = 0.033) were significant (Table 4). The effect of body fat percentage change was then examined for each menopausal group separately. For premenopausal women (*n* = 86), body-fat percentage change was not associated with either distant metastasis or all-cause mortality. However, for postmenopausal women, higher gain in body-fat percentage was associated with higher risk of disease metastasis (HR = 1.3, *p* = 0.035) and marginally associated with higher risk of all-cause mortality (HR = 1.5, *p* = 0.091). For covariate effects, disease stage was consistently found to be a significant predictor on metastasis and mortality for both pre- and postmenopausal women (*p* < 0.001). Increased in age was associated with lower risk of disease metastasis (HR = 0.9, *p* = 0.029) in pre-menopausal women only (Table 5).

**Table 4:** The interaction effect of body fat percentage change and menopausal status on distant metastasis and all-cause mortality in women with breast cancer (*N* = 131)

	*B (± SE)*	Hazard ratio (95% CI)	*p*
Metastasis			
Advanced disease (Stage III)	2.05 (± 0.49)	7.80 (3.00–20.28)	< .001
ER positive	0.87 (± 0.58)	2.39 (0.76–7.45)	.134
PR positive	−0.75 (± 0.52)	0.47 (0.17–1.30)	.146
Age	−0.03 (± 0.03)	0.97 (0.93–1.02)	.235
Regular exercise	0.01 (± 0.52)	1.01 (0.37–2.82)	.978
Body fat % change × menopausal status	0.29 (± 0.11)	1.33 (1.06–1.66)	.012
Death			
Advanced disease (Stage III)	3.22 (± 0.79)	25.09 (5.34–117.89)	< .001
ER positive	0.41 (± 0.74)	1.50 (0.35–6.45)	.585
PR positive	−0.77 (± 0.66)	0.46 (0.13–1.70)	.246
Age	0.02 (± 0.03)	1.02 (0.96–1.09)	.555
Regular exercise	−0.32 (± 0.72)	0.73 (0.18–2.98)	.660
Body fat % change × menopausal status	0.32 (± 0.15)	1.18 (1.03–1.86)	.033

Body fat % change = body fat percentage at 6 months after surgery minus body fat percentage before surgery

*ER* estrogen receptor, *PR* progesterone receptor

**Table 5:** Effect of body fat percentage change on distant metastasis and survival by menopausal status in women with breast cancer

	Premenopausal (*n* = 86)			Postmenopausal (*n* = 45)		
	*B (± SE)*	HR (95% CI)	*p*	*B (± SE)*	HR (95% CI)	*p*
Metastasis						
Advanced disease (Stage III)	1.9 (± 0.6)	6.4 (1.83–22.64)	.004	2.5 (± 1.0)	11.7 (1.55–88.07)	.017
ER positive	1.7 (± 0.9)	5.5 (0.86–35.11)	.071	−0.3 (± 0.9)	0.8 (0.13–4.59)	.781
PR positive	−1.3 (± 0.8)	0.3 (0.06–1.22)	.088	0.3 (± 0.9)	1.4 (0.21–8.70)	.749
Age	−0.1 (± 0.1)	0.9 (0.83–0.99)	.029	0.1 (± 0.1)	1.0 (0.90–1.12)	.940
Regular exercise	−0.4 (± 1.1)	0.7 (0.08–6.00)	.739	0.1 (± 0.7)	1.1 (0.24–4.57)	.945
Body fat % change	0.1 (± 0.1)	1.1 (0.95–1.21)	.278	0.3 (± 0.1)	1.3 (1.02–1.72)	.035
Death						
Advanced disease (Stage III)	3.2 (± 1.0)	24.7 (3.77–161.63)	.001	4.7 (± 1.9)	106.5 (2.78–4084.85)	.012
ER positive	2.1 (± 1.3)	8.6 (0.71–103.50)	.091	−0.9 (± 1.3)	0.4 (0.03–4.88)	.476
PR positive	−2.0 (± 1.1)	0.1 (0.02–1.14)	.067	−0.5 (± 1.4)	0.6 (0.04–10.19)	.749
Age	−0.1 (± 0.1)	0.9 (0.87–1.11)	.769	0.1 (± 0.1)	1.1 (0.92–1.29)	.298
Regular exercise	0.3 (± 1.2)	1.3(0.13–13.24)	.815	−1.1 (± 1.1)	0.3(0.04–2.75)	.312
Body fat % change	0.1 (± 0.1)	1.1(0.95–1.33)	.188	0.4 (± 0.2)	1.5(0.94–2.25)	.091

*BMI* body mass index*, ER* estrogen receptor, *PR* progesterone recepto*r, CI confidence interval*

## Discussion

Our study contributes to knowledge on the effect of obesity on breast cancer outcomes by clarifying that the effect of weight gain previously reported [6–8] is due to changes in body fat composition after breast cancer surgery. After adjusting for the effects of covariates, we found that increased body-fat percentage predicted distant metastasis in postmenopausal women after breast cancer surgery, but not in premenopausal women.

Many previous studies have demonstrated the association between obesity and risk of disease recurrence [11, 34] or mortality [6, 13, 35, 36] in breast cancer women. However, still other studies failed to find this association [7, 37]. The way obesity was measured in these studies included weight/BMI, waist-hip ratio, or weight gain after diagnosis. None of these studies used the direct measure of body fat to represent obesity. In addition, none of these studies considered the potential role of menopausal status. In the current study, we directly examined the relationship between body-fat percentage change and the disease progression. When menopausal status was not considered in the model, the relationship between body-fat percentage gain and the metastasis/mortality was only marginal which did not reach statistical significance of 0.05. However, when breast cancer women were divided into pre- and post-menopausal groups, the effect of body fat percentage gain was found to be significant in predicting metastasis in postmenopausal women but not in premenopausal women. Therefore, it is suspected that the inconsistent findings in previous studies may be due to indirect measures of obesity and lack of consideration of menopausal status.

Our findings suggest that menopausal status moderates the link between body-fat gain and disease metastasis in breast cancer women. Gain in body-fat percentage after breast cancer surgery was a predictive factor only for postmenopausal women. This finding may be explained by different sources of estrogen production in premenopausal and postmenopausal women. For premenopausal women, the ovaries are the main source of estrogen production, whereas adipose tissue serves as the extragonadal source of estrogen for postmenopausal women [38]. In postmenopausal women, adipose tissue contains aromatase that can convert androgen to estrogen [38]. Therefore, increased body fat in these women may increase estrogen concentrations, consequently leading to a poorer outcome [39]. In addition, obese postmenopausal women have lower serum levels of sex hormone-binding globulin (SHBG) than obese premenopausal women [10]. SHBG can bind and inhibit estradiol, a kind of estrogen-like hormone. Lower SHBG levels will result in increased levels of circulating unbound estrogen that may promote tumor progression [10].

Based on our study findings, we recommend that postmenopausal women with breast cancer maintain or decrease their body fat during or after treatment. However, the traditional clinical practice is to advise patients to maximize caloric intake to ensure a good physical condition against the toxic effects of adjuvant chemotherapy [10]. Based on our findings, we think this traditional diet suggestion may not be valid for all cancer types. The diet suggestion for breast cancer survivors may need to modify, at least for postmenopausal women. Future diet or exercise interventions that aim to facilitate body fat loss and retain lean body mass may be considered.

This study has several limitations. First, the BIA machine (RJL Systems, Clifton Township, MI, USA) used in this study may be less accurate in Asian women than in western women, who have relatively longer limbs. This difference may underestimate body-fat percentage in shorter limbed persons like Asian women [40], despite a report that the BIA machine is easy to use and has good accuracy in detecting changes in women’s body composition [30]. Second, the gain in body fat percentage was defined by the difference between measures at pre-surgery and 6 months post-surgery. Some women may recover from the temporary weight or body fat gain caused by the cancer treatment. Third, 3–5% of the data on disease outcomes were missing due to patients being lost to follow-up. These patients may have had worse or better disease outcomes than study participants. Fourth, the accuracy of this study’s predictive ability may have been affected by its high censoring rate (82.4% for distant metastasis; 90.1% for all-cause mortality), reflecting that time to distant metastasis or all-cause mortality was unavailable due to participants lost to follow-up or the outcome event not occurring before study end [41]. Finally, the findings of this study are limited to the women recruited from one hospital and cannot be generalized to all women with breast cancer in Taiwan.

## Conclusions

This study highlights the role of increased body fat percentage after breast cancer treatment on disease progression in postmenopausal women. We suggest periodically measuring not only body weight but also body fat and monitoring changes in body fat percentage. More research is needed on the effect of changes in body fat on breast cancer survival in women with different menopausal status.

## Abbreviations

BIA: Bioelectrical impedance analyzer
BMI: Body mass index
BW: Body weight
DXA: Dual X-ray absorptiometry
ER: Estrogen receptors
HR: Hazard ratio
PR: Progesterone receptors
SHBG: Sex hormone-binding globulin
